# The gray matter atrophy and related network changes occur in the higher cognitive region rather than the primary sensorimotor cortex after spinal cord injury

**DOI:** 10.7717/peerj.16172

**Published:** 2023-10-09

**Authors:** Xin Chen, Ling Wang, Weimin Zheng, Yanhui Yang, Beining Yang, Yongsheng Hu, Jubao Du, Xuejing Li, Jie Lu, Nan Chen

**Affiliations:** 1 Department of Radiology and Nuclear Medicine, Xuanwu Hospital, Capital Medical University, Beijing, China; 2 Beijing Key Laboratory of Magnetic Resonance Imaging and Brain Informatics, Beijing, China; 3 Department of Functional Neurosurgery, Xuanwu Hospital, Capital Medical University, Beijing, China; 4 Department of Rehabilitation Medicine, Xuanwu Hospital, Capital Medical University, Beijing, China; 5 Department of Radiology, China Rehabilitation Research Center, Beijing, China

**Keywords:** Spinal cord injury, Gray matter volume, Functional connectivity, Cognitive function, Voxel-based morphometry

## Abstract

**Objective:**

This study used functional magnetic resonance imaging (fMRI) to explore brain structural and related network changes in patients with spinal cord injury (SCI).

**Methods:**

Thirty-one right-handed SCI patients and 31 gender- and age-matched healthy controls (HC) were included. The gray matter volume (GMV) changes in SCI patients were observed using voxel-based morphometry (VBM). Then, these altered gray matter clusters were used as the regions of interest (ROIs) for whole-brain functional connectivity (FC) analysis to detect related functional changes. The potential association between GMV and FC values with the visual analog scale (VAS), the American Spinal Injury Association (ASIA) score, and the course of injuries was investigated through partial correlation analysis.

**Results:**

GMV of the frontal, temporal, and insular cortices was lower in the SCI group than in the HC group. No GMV changes were found in the primary sensorimotor area in the SCI group. Besides, the altered FC regions were not in the primary sensorimotor area but in the cingulate gyrus, supplementary motor area, precuneus, frontal lobe, and insular. Additionally, some of these altered GMV and FC regions were correlated with ASIA motor scores, indicating that higher cognitive regions can affect motor function in SCI patients.

**Conclusions:**

This study demonstrated that gray matter and related network reorganization in patients with SCI occurred in higher cognitive regions. Future rehabilitation strategies should focus more on cognitive functions.

## Introduction

Spinal cord injury (SCI) is a highly disabling disease (GBD 2016 Traumatic Brain Injury and Spinal Cord Injury Collaborators, 2019; Quadri et al., 2020), and its sensorimotor dysfunction affects the quality of life and rehabilitation of patients. However, several physiological and psychological studies have shown that cognitive deficits and neuropathic pain are highly prevalent in SCI patients (Burke et al., 2017; Lim et al., 2017) and also hinder the rehabilitation process and effectiveness of those patients returning to society (Awad et al., 2020; Murray et al., 2007; Quadri et al., 2020; Sachdeva et al., 2018; Williams & Murray, 2015). The cognitive deficits of SCI, such as sensory (Lenggenhager et al., 2012) and visual (Pernigo et al., 2012; Scandola et al., 2020) proprioceptive disturbance, premotor dysfunction (Chiaravalloti et al., 2020), and information processing speed (Chiaravalloti et al., 2020), may hinder basic care, functional recovery, and daily life. Moreover, cognitive impairment or pain state may cause depression, which in turn aggravates cognitive impairment and hinders psychosocial adaptation to disability (Brakel & Hook, 2019; Li et al., 2020c).

Some neuroimaging findings provide evidence for the influence of psychological aspects on patients with SCI. For example, the gray matter volume (GMV) changes in these corresponding regions have been found in SCI patients. These regions, including cingulate cortex, cerebellar cortex, insular cortex, and prefrontal cortex, are associated with emotional, cognitive, and neuropathic pain (Chen et al., 2017; Freund et al., 2013; Hou et al., 2014; Jutzeler et al., 2016; Li et al., 2020b; Nicotra et al., 2006; Rolls et al., 2020a; Wrigley et al., 2009a; Wrigley et al., 2009b; Yoon et al., 2013).

However, the specific mechanisms underlying these changes in regions remain controversial. In some studies, for example, cingulate cortex atrophy is thought to be caused by changes in cognitive and emotional processing due to disuse-induced transneuronal degeneration (Chen et al., 2017; Wrigley et al., 2009a), while it is thought to be caused by pain in others (Jutzeler et al., 2016; Yoon et al., 2013). This divergence may be because a structure can be a component of different networks. In addition, GMV changes in the right orbitofrontal cortex and dorsolateral anterior cingulate cortex, which are not classical sensorimotor areas, correlate with motor functions (Chen et al., 2017). This indicates a specific connection between them and the sensorimotor area. Therefore, the results of brain reorganization may be better interpreted in terms of the network where the structure may be involved. However, few studies have used altered regions as the areas of interest (ROIs) for whole-brain functional network analysis, all of which are used on patients with incomplete SCI (Chen et al., 2018; Li et al., 2020a). In addition, the existence of gray matter reorganization of primary sensorimotor areas in SCI patients is controversial (Nardone et al., 2018).

This study aimed to investigate the regions where the brain changes after SCI and their role in brain networks. First, the data were processed at the whole-brain level, and the voxel-based morphometry (VBM) method was used to determine regions and whether and where the GMV changed. The result was presented on the automated anatomical labeling (AAL) atlas. Second, the GMV regions were defined as the ROIs to explore the possible role of the altered GMV regions in brain network reorganization. If the GMV area covered more than one region of AAL, the GMV area was further segmented into subregions according to the AAL atlas to distinguish the specific role of each subregion. Then, a seed-based resting-state functional connectivity (FC) analysis was adopted to find where the FC changed with them, based on the notion that aberrant network nodes can result in aberrant signaling and then spread to the whole network or subnetworks (Menon, 2011). Finally, the possible association between the GMV changes as their corresponding FC changes and clinical variables was assessed to further demonstrate their role in brain reorganization.

## Materials & Methods

### Subjects

Thirty-one right-handed patients with SCI (24 males and seven females, with a range of 22-71 years old and a mean age of 47.3  ± 13.46 years) were included in this study. All the patients suffered bilateral sensorimotor dysfunction and exhibited hyper-intensity at the injury level on conventional T2-weighted magnetic resonance imaging (MRI). There was no brain lesion confirmed by conventional MRI, no limb trauma or brachial plexus injury, no symptoms associated with traumatic brain injury such as dizziness, headache, and loss of consciousness, and no pain relief treatment after SCI. According to the American Spinal Injury Association (ASIA) impairment scale, 11 were classified as grade A, 5 as grade C, and 15 as grade D (Walden et al., 2016). The mean course of the disease was 0.58 (0.17, 7.00) years.

A clinical assessment was conducted on all the patients, including sensory score (light touch and pinprick) and motor score according to the ASIA score (Marino et al., 2003), as well as pain score according to the visual analog scale (VAS) before MRI acquisition. The specific clinical assessment was consistent with previous studies (Chen et al., 2019; Li et al., 2020b). Table 1 contains detailed information on these patients.

**Table 1:** Clinical data for the SCI patients.

ID	Age (years)	Sex	Course (years)	Level of lesion^a^	ASIA scale	ASIA pinprick score L/R	ASIA light touch score L/R	ASIA motor (L)	ASIA motor (R)	VAS
1	55	F	0.75	C 3-4	D	56/56	56/56	46	50	6
2	53	M	0.02	C 4-5	D	51/51	51/51	45	45	0
3	34	F	1.0	L 1	D	46/46	46/46	47	47	4
4	38	M	0.08	T 12	A	46/40	46/40	37	37	6
5	28	F	0.58	L 1	D	45/45	45/45	35	35	0
6	47	M	0.01	C 4	D	50/50	50/50	47	47	0
7	55	M	9.0	L 3	A	39/48	39/48	25	25	9
8	42	M	9.0	T 12	A	45/45	45/45	28	25	9
9	38	M	7.0	T 12	A	37/37	37/37	31	25	9
10	40	F	12.0	L 1-2	D	43/42	43/42	45	45	7
11	48	M	0.83	T 10	C	54/54	54/54	27	27	0
12	52	M	0.25	T 10	A	28/28	28/28	25	25	0
13	50	M	1.0	C 5-7	A	37/37	37/37	17	17	9
14	33	M	0.1	L 1	D	50/50	50/50	29	29	0
15	65	F	0.17	T 4-10	D	56/56	56/56	40	40	0
16	61	M	0.08	C 3-4	D	55/55	55/55	43	43	0
17	60	M	3.0	C 3-7	C	56/29	56/29	50	20	9
18	71	M	1.0	C 3-4	D	51/51	51/51	30	30	5
19	56	M	33	C 4	C	31/56	31/56	15	25	9
20	67	M	3.0	L 1-4	C	38/38	38/38	25	35	6
21	52	M	11.0	L 1	A	38/38	38/38	25	25	6
22	57	M	7.0	C 5	D	38/38	38/38	45	40	6
23	23	M	0.5	T 8	D	42/42	42/42	45	45	7
24	40	M	0.2	T 4	A	21/21	21/21	25	25	0
25	23	F	0.42	T 8	D	51/47	51/47	45	45	6
26	48	M	0.2	C 3-6	A	56/56	56/56	18	20	0
27	66	F	0.17	T 8	C	42/42	42/42	45	45	0
28	31	M	0.17	T 9	D	44/44	44/44	45	40	0
29	58	M	29.0	T 12	A	36/36	36/36	25	25	8
30	52	M	0.03	C 2-6	A	25/13	25/13	4	0	0
31	22	M	0.01	C 6	D	50/50	50/50	50	50	0

**Notes.**

a The level refers to the lesion on conventional T2-weighted MR.

ASIA American Spinal Injury Association L left R right VAS visual analogue scale

To investigate whether the level of injury, completeness of injury, presence of pain, and course of injury affect the GMV or FC differences, the SCI group was further divided into cervical SCI (12 cases) and sub-cervical SCI (19 cases) sub-groups, into complete SCI (11 cases) and incomplete SCI (20 cases) sub-groups, into painful SCI (17 cases) and pain-free SCI (14 cases) sub-groups, and into acute SCI (<1 year; 18 cases) and chronic & extended-chronic SCI (≥1 year; 13 cases) sub-groups (Macklin et al., 2017), respectively.

Thirty-one healthy right-handed volunteers (20 males and 11 females, with a range of 25–65 years old and a mean age of 46.7 ± 11.8 years) were recruited as the healthy controls (HC) group.

This study protocol received approval from the Ethics Committee of Xuanwu Hospital, Capital Medical University (Ethics No: (2020) 003). Each participant was provided written informed consent in accordance with the Declaration of Helsinki.

### Image acquisition

Images were acquired with a 12-channel phase-array head coil using a 3.0 T MRI system (Trio Tim, Siemens, Erlangen, Germany). Earplugs were used to reduce imaging noise, while tight but comfortable foam padding was used to reduce head motion. Participants were asked to close their eyes, stay awake, breathe evenly, and try to avoid specific thoughts. Visible brain abnormalities were excluded by scanning a traditional brain axial fluid-attenuated inverse recovery sequence. A 3D magnetization-prepared rapid gradient-echo sequence (MP-RAGE) was used to acquire 3D high-resolution structural T1-weighted images in sagittal direction with the following parameters: voxel size = 1 ×1 ×1 mm^3^; matrix = 256 ×256; field of view (FOV) = 256 ×256 mm^2^; slice thickness = one mm; number of slices = 192; flip angle (FA) = 9°; inversion time (TI) = 1100 ms; echo time (TE) = 2.13 ms; and repetition time (TR) = 1800 ms. Additionally, an echo-planar imaging sequence was used to acquire resting-state functional images with the following parameters: voxel size = 3.4 ×3.4 ×3.0 mm^3^; matrix = 64 ×64; FOV = 220 ×220 mm^2^; FA = 90°; TE = 30 ms; TR = 2000 ms; slice thickness = three mm; number of slices = 35; and inter-slice gap = one mm.

### Post-processing

### VBM analysis

Three-dimensional T1-MP-RAGE images were post-processed using SPM12 software in MATLAB 2021a (MathWorks, Natick, MA, USA), which was consistent with the previous study (Chen et al., 2017). The result was presented based on the AAL atlas.

### FC analysis

Functional images were pre-processed and statistically analyzed using SPM12. To reach the magnetization equilibrium, the first 10 volumes of each functional time series were discarded. The remaining 170 images were corrected for the time delay between different slices and realigned to the first volume. Head position records were provided by computing head motion parameters based on the translation estimation in each direction and angular rotation on each axis for each volume. The realigned images were spatially normalized to the Montreal Neurological Institute (MNI) EPI template and resampled to a 2-mm cubic voxel. The subjects exhibited head motion parameters below the 2° rotation and 2-mm translation threshold in the *x*-, *y*-, and *z*-directions. Linear regression was used to remove several spurious variance sources, such as mean BOLD signals in the ventricle and white matter regions, global mean BOLD signals, linear drift, and estimated motion parameters. The time series of each voxel was temporally band-pass filtered (0.01–0.08 Hz) to minimize the effects of high-frequency noise and low-frequency drift. Finally, the images were smoothed with a half-maximum Gaussian kernel with a full width of six mm.

An ROI-based method was used to determine the resting-state FC. ROIs, as the brain regions where the GMV displayed significant differences between the two groups, were extracted. If the GMV area covered more than one region of AAL, the GMV area was further segmented into subregions according to the AAL atlas. Then, the FC of each sub-region was calculated. Fisher’s r-to-z transformation was used to compute correlation coefficients between the mean time series and convert them to z-values for each subject. The REST software was used to improve normality. Subsequently, individual values were tested using a random effect one-sample *t*-test to determine the brain regions significantly positively correlated with the ROI regions. Intergroup comparisons were only performed in the brain regions of each group.

### Statistical analysis

A two-sample *t*-test was used to assess the age and sex between the two groups. Fisher exact test was used to assess ASIA sensory and motor scores between the two sides. Based on the GLM in SPM, a voxel-wise two-sample *t*-test was used to compare GMV and FC differences between the two groups, with age and sex as nuisance covariates (voxel-level uncorrected *p* < 0.0001, non-stationary cluster-level FWE correction with *p* < 0.05).

The mean GMV and FC values of each cluster with statistical significance between the two groups were extracted for SCI subgroup analysis. If a cluster covered more than one label of AAL, the cluster was further segmented into subregions according to the AAL atlas. The inter-subgroup differences were measured using a two-sample *t*-test, with age and sex as nuisance covariates. To investigate the potential association between GMV and FC values with clinical variables (ASIA score, VAS, and the course of injuries) in the whole SCI group, a partial correlation analysis was performed using MATLAB 2021a. The Rule of Thumb was used to interpret correlation coefficients. The coefficients ranging from 0.1 to 0.39, 0.4 to 0.69, 0.7 to 0.89, and 0.9 to 1.0 indicate a weak, moderate, strong, and very strong correlation, respectively (Schober, Boer & Schwarte, 2018).

## Results

There was no statistically significant difference between the HC group and the SCI group regarding age (*p* = 0.85) and sex (*p* = 0.24). In addition, no statistically significant differences were observed in the sensory score (*p* = 0.70 for both pinprick and touch scores) and motor score (*p* = 0.53 for both upper and lower extremities) between the left and right sides.

### GMV and related resting-state FC changes in the SCI group

**Figure 1:**
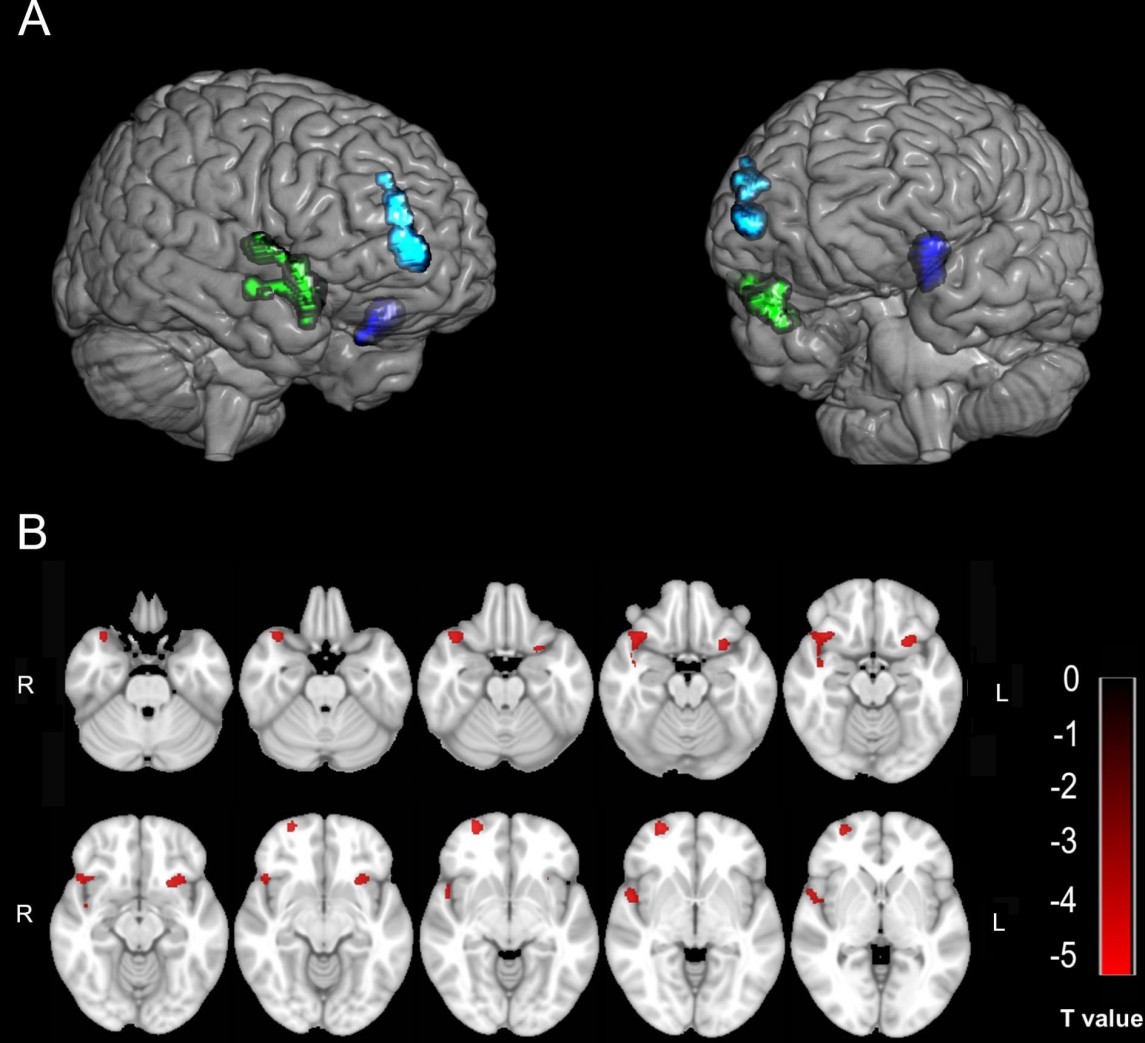
The decreased GMV in the SCI group compared with the HC group. (A & B) Three-dimensional and two-dimensional locations of these regions, respectively. The color bar in B represents the T value.

Compared with the HC group, the SCI group showed three decreased GMV clusters in the right temporal pole in the superior temporal gyrus (TPOsup)/the right pars orbitalis in the inferior frontal gyrus (IFGorb), the pars orbitalis in the right superior frontal gyrus (SFGorb)/the right superior frontal gyrus (SFG)/the right middle frontal gyrus (MFG)/the right medial orbital in the superior frontal gyrus (PFCventmed), and the insular (INS)/the left IFGorb (Table 2; Fig. 1). No GMV changes were found in the primary sensorimotor area in the SCI group. The FC of each sub-cluster was calculated. For example, the right TPOsup/right IFGorb cluster was segmented into two sub-clusters (the right TPOsup and the right IFGorb), and then the FC of each sub-cluster was calculated.

**Table 2:** Regions with decreased gray matter volume in patients (voxel-level uncorrected *p* < 0.0001, non-stationary cluster-level FWE correction with *p* < 0.05).

**Clusters**	**MNI coordinates**			**Peak *T* value**	**Cluster size (voxels)**
	X	Y	Z		
TPOsup_R/IFGorb_R	40.5	18	−18	−5.11	860
SFGorb_R/SFG_R/MFG_R/PFCventmed_R	27	60	−6	−5.40	419
INS_L/IFGorb_L	−28.5	16.5	−16.5	−5.07	347

**Notes.**

MNI Montreal Neurological Institute L left R right TPOsup temporal pole in the superior temporal gyrus IFGorb pars orbitalis in the inferior frontal gyrus SFGorb pars orbitalis in superior frontal gyrus SFG superior frontal gyrus MFG middle frontal gyrus PFCventmed the medial orbital in the superior frontal gyrus INS insular

**Figure 2:**
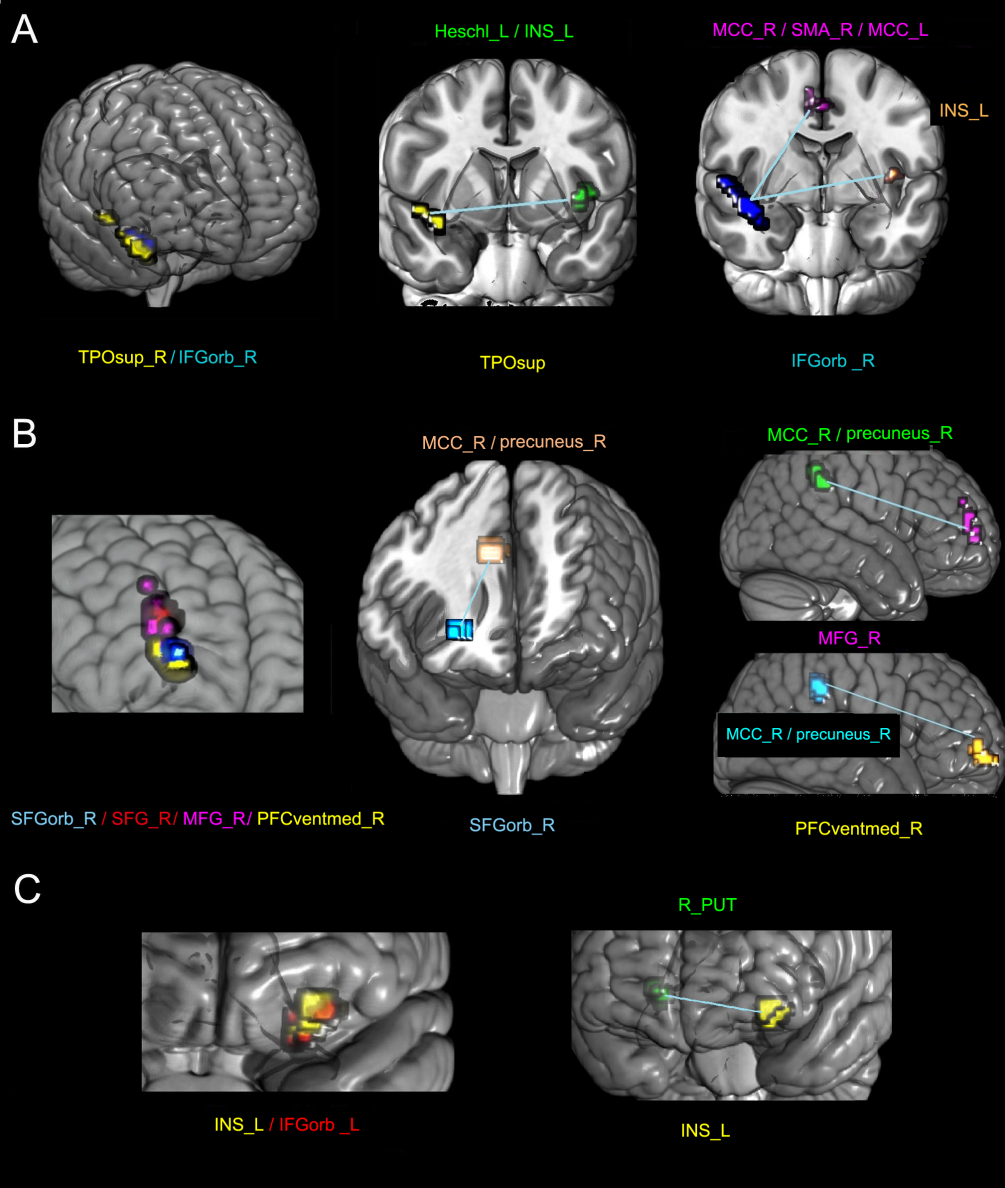
The altered FC between different sub-clusters with the whole brain in the SCI group compared with the NC group. The left columns of A, B, and C represent three clusters with decreased GMV. Different colors represent different sub-clusters in each panel. The other columns represent the areas of the statistically altered FC between different sub-clusters with the whole brain in the SCI group compared with the HC group. In each panel, the sub-clusters with the same color are the same. L, left; R, right; TPOsup, pars orbitalis in the inferior frontal gyrus; IFGorb_R, pars orbitalis in the inferior frontal gyrus; INS, insular; MCC, middle cingulate; SMA, supplementary motor area; SFGorb, pars orbitalis in the superior frontal gyrus; MFG, middle frontal gyrus; SFG, superior frontal gyrus; PFCventmed, medial orbital in the superior frontal gyrus; and PUT, putamen.

The altered FC regions with these areas were also not in the primary sensorimotor area but in the cingulate gyrus, supplementary motor area (SMA), precuneus (precuneus), frontal lobe, and INS. Table 3 and Fig. 2 show the detailed results.

**Table 3:** Voxel-based FC analysis of the regions with decreased GMV in patients (voxel-level uncorrected *p* < 0.0001, non-stationary cluster-level FWE correction with *p* < 0.05).

**Cluster**	**Sub-cluster**	**FC regions**	**MNI coordinates**			**Peak *T* value**	**Cluster size (voxels)**
			X	Y	Z		
TPOsup_R/IFGorb_R	TPOsup_R	Heschl_L/INS_L	−42	−15	6	−5.44	17
	IFGorb _R	MCC_R/SMA_R/MCC_L	0	−6	45	−4.94	30
		INS_L	−39	−15	6	−5.42	13
SFGorb_R/SFG_R/	SFGorb_R	MCC_R/precuneus_R	6	−39	39	−5.09	19
MFG_R/PFCventmed_R	SFG_R	None					
	MFG_R	MCC_R/precuneus_R	6	−45	48	−5.30	49
	PFCventmed_R	MCC_R/precuneus_R	6	−39	39	−5.78	34
INS_L/IFGorb _L	INS_L	R_PUT	21	15	−3	−5.06	14
	IFGorb_L	None					

**Notes.**

MNI Montreal Neurological Institute ROI region of interest L left R right TPOsup the pars orbitalis in the inferior frontal gyrus IFGorb the pars orbitalis in the inferior frontal gyrus INS insular MCC middle cingulate SMA supplementary motor areas SFGorb pars orbitalis in superior frontal gyrus SFG superior frontal gyrus MFG middle frontal gyrus PFCventmed medial orbital in the superior frontal gyrus PUT Putamen

### Differences between different SCI sub-groups

The GMV of the sub-cluster right MFG in the incomplete sub-group was significantly larger than that in the complete sub-group (*p* = 0.04). The GMV of the left INS/the left IFGorb in the pain-free sub-group was significantly larger than that in the painful sub-group (*p* = 0.02).

No other significant differences in GMV and FC values were observed between pain-free and painful sub-groups, cervical and sub-cervical SCI sub-groups, complete and incomplete sub-groups, and acute and chronic & extended-chronic sub-groups.

### Correlation between GMV and FC values and clinical variables in the SCI group

Among all clinical indicators, the GMV and the FC value were associated with the ASIA motor score, and the FC value was related to the the disease course. No statistical correlations existed between the VAS and either the GMV or the FC value.

The ASIA motor score of the left extremity: (1) it had a moderate positive correlation with the GMV of the right TPOsup/the right IFGorb (*r* = 0.53, *p* < 0.01), as well as its sub-cluster right TPOsup (*r* = 0.44, *p* = 0.02) and the right IFGorb (*r* = 0.51, *p* = 0.01); (2) it had a moderate positive correlation with the FC between the sub-cluster right SFGorb with the right middle cingulate (MCC)/the right precuneus (*r* = 0.42, *p* = 0.02), and the FC between the sub-cluster right PFCventmed with the right MCC/the right precuneus (*r* = 0.40, *p* = 0.03), respectively; (3) it had a weak positive correlation with the GMV of the right SFGorb/the right SFG/the right MFG/the right PFCventmed (*r* = 0.38, *p* = 0.04).

The ASIA motor score of the right extremity had a moderate positive correlation with the FC between the right MFG and the right precuneus/the right MCC (*r* = 0.43, *p* = 0.02).

The course of injury had a moderate correlation with the FC between the right IFGorb with the right MCC/the right SMA/the left MCC (*r* = 0.43, *p* = 0.02).

The correlation analysis curves of the above clinical variables are presented in Fig. S1.

We further examined the relationship between GMV and corresponding FC in different subgroups and clinical variables. Due to the limited sample size of subgroups, we exclusively analyzed those that showed statistical differences in GMV. Specifically, we investigated the correlation between the GMV of the right MFG sub-cluster and the corresponding FC with clinical indicators in the complete and incomplete subgroups and the GMV of the left INS/the left IFGorb between the pain and pain-free subgroups. The main difference between the entire group and a subgroup is that the FC between the sub-cluster of the right MFG and the right MCC/the right precuneus of the complete subgroup also had positive correlations to the ASIA motor score of the left extremity (*r* = 0.62, *p* = 0.04). Additionally, both the sub-cluster left INS and the sub-cluster left IFGorb in the pain subgroup showed positive correlations with the ASIA motor score of the left (*r* = 0.64, *p* = 0.01) and right (*r* = 0.61, *p* = 0.01) extremity. Detailed results are presented in Table S1.

## Discussion

This study demonstrated frontal, temporal, and insular cortex atrophy in the SCI group. Furthermore, the altered FC regions with these atrophic cortices occurred in the cingulate gyrus, SMA, precuneus, frontal lobe, and insular. No changes of GMV or FC occurred in the primary sensorimotor area. Moreover, some of the altered GMV and FC regions were correlated with ASIA motor scores, indicating that the areas outside the primary sensorimotor cortex also affect motor function in SCI patients.

### Possible altered networks in the atrophic frontal, temporal, and insular cortex

### The right central executive network (CEN)

Three atrophic frontal GMV sub-clusters (the right SFGorb, MFG, and PFCventmed) had decreased FC with the right cingulate and precuneus (the right MCC/the right precuneus). The anatomical location of the GMV sub-cluster and its relative altered FC region belongs to the right CEN. Many higher-order executive tasks that require consciously directed and focused attention, such as decision-making, motor planning, and working memory, depend on the CEN. These function declines are indeed found in SCI patients (Chiaravalloti et al., 2020; Huang et al., 2017; Macciocchi, Seel & Thompson, 2013), providing evidence for this inference. All of the FC positively correlate with ASIA motor score, indicating that the altered network between the right frontal lobe with the right cingulate and precuneus is related to motor function. This may suggest that the impairment of the CEN network in SCI patients is due to motor dysfunction. In addition to its correlation with the ASIA motor score of the right extremity of the entire group, both complete and incomplete subgroups, the FC between the sub-cluster of the right MFG and the right MCC/the right precuneus of the complete sub-group is also associated with the ASIA motor score of the left extremity. This indicates contralateral compensation in the CEN when the efferent motor pathway is completely interrupted, which will be further explored by expanding the sample size.

### The right frontal aslant tract (FAT)

The atrophic GMV in the right IFGorb had decreased FC with the right SMA and the bilateral cingulate. It covers the right FAT connecting the IFG and SMA (Vergani et al., 2014). The right FAT is thought to be associated with inhibitory control, suppressing the cortical output for behaviors that conflict with a goal or target behavior (Dick et al., 2019). Since the output in SCI patients is partially or entirely lost, the decreased GMV or FC value in these regions is likely to reflect disuse-induced motor degeneration, which is consistent with the results that the GMV of the sub-cluster right IFGorb and its relative FC correlated with the ASIA motor score. Besides, since the right IFG and orbitofrontal cortex are involved in converting negative emotional experiences into motor actions (Rolls et al., 2020a; Rolls, Cheng & Feng, 2020b) and send information to the cingulate (Vogt, 2016) and SMA (Vergani et al., 2014), the reduced GMV or FC valve in these regions may be caused by depression and emotional stress after SCI and then interfere with the desired behaviors. The findings that the FC between the frontal lobe with the bilateral cingulate and the right SMA had a moderate correlation with the course of injury supports the idea that cognitive dysfunction is progressively worsening in SCI patients (Molina et al., 2018). The right FAT should be concerned in future studies of the mechanisms for how the psychological aspect induced by SCI affects brain reorganization to provide possible targets for more effective treatment.

### The salience network (SN)

The decreased GMV regions of the frontal, temporal, and insular cortices and the altered FC between the sub-cluster and the insular cortex belong to the core hub of the SN (Uddin, 2015). This network integrates internal and external sensory information to generate neural responses (Seeley et al., 2007), guiding behavior by identifying the most subjective salience among many internal and external stimuli at one time (Chen et al., 2016) and switching activity between the CEN and the default mode network (Chen et al., 2016). The function of the right FAT is similar to the SN, further demonstrating that the atrophy of the right OFC is related to disturbed target behaviors. The result that the GMV of the left INS/the left IFGorb in the pain-free sub-group was significantly larger than that in the painful sub-group, which is consistent with the previous study (Seeley et al., 2007), indicates that the interfered SN is associated with pain. It could be supported by the view that the SN is a pain matrix of the brain where the central pain is considered a behavioral response to the imbalance of internal and external sensory information (Legrain et al., 2011). The moderate positive correlations between the ASIA motor score of the bilateral extremities and both the sub-cluster INS_L and the sub-cluster IFGorb_L in the pain sub-group provide further support for a connection between pain and motor function through the SN.

### The possible reasons for no GMV or FC changes in the primary sensorimotor cortex

The negative finding in the primary sensorimotor cortex is consistent with some neuroimaging studies. Based on the results of this study, the negative finding further supports the inference that the persistent statement of pain and/or depression is more remarkable in brain neurodegeneration than the sensorimotor aspect after SCI. The GMV atrophy in the primary sensorimotor cortex is thought to be caused by Wallerian degeneration (Wrigley et al., 2009a) or disuse-induced transneuronal degeneration (Bose et al., 2005; Jurkiewicz et al., 2006). Either of them may not be as strong as the effect exerted on brain networks by the persistent condition of negative emotions or pain (Wu et al., 2014a; Wu et al., 2014b). This significance may be further exacerbated by differences in post-processing methods. The VBM method of this study was based on the whole-brain level, which means not focusing on some specific regions due to the aim of finding brain reorganization without any prior hypotheses. In addition, a stringent primary threshold was used to avoid problems in multiple comparisons (Woo, Krishnan & Wager, 2014). Therefore, small changes in the sensorimotor cortex may make it difficult to reach statistical significance using the threshold and whole-brain level analysis.

Several limitations should be addressed in the present study. Firstly, due to the limited sample size, there was heterogeneity in the level and degree of SCI, the presence and degree of pain, and the course of injury in SCI patients. To minimize the effects of heterogeneity, patients were divided into different subgroups according to the above aspects, and inter-subgroup differences were compared, respectively. Future studies with a larger sample size will focus on similar types of patients. Secondly, neuropsychological measurements were not included in this study. Corresponding evaluations will be developed in future studies of the circuits discovered in the current study to further understand brain reorganization after SCI. Thirdly, a longitudinal study will be necessary to explore the dynamic change of reorganization in the future.

## Conclusions

This study demonstrates that gray matter and related network reorganization in patients with SCI occur in higher cognitive regions. Besides, the negative finding in the primary sensorimotor cortex supports the inference that the persistent statement of pain and/or depression is more remarkable in brain neurodegeneration than the sensorimotor aspect after SCI. This provides new ideas for brain reorganization after SCI. Future rehabilitation strategies should pay more attention to cognitive functions.

##  Supplemental Information

10.7717/peerj.16172/supp-1 Figure S1 Correlation between GMV and FC values and clinical variables in the SCI group L, left; R, right; SMA, supplementary motor area; IFGorb, pars orbitalis in the inferior frontal gyrus; TPOsup, pars orbitalis in the inferior frontal gyrus; MCC, middle cingulate; SFGorb, pars orbitalis in the superior frontal gyrus; MFG, middle frontal gyrus; and PFCventmed, medial orbital in the superior frontal gyrus.

10.7717/peerj.16172/supp-2 Table S1 Correlation between GMV and FC values and clinical variables in the SCI subgroups that showed statistical differences in GMV Note: ASIA, American Spinal Injury Association; L, left; R, right; MFG, middle frontal gyrus; MCC, middle cingulate; INS, insular; and IFGorb, the pars orbitalis in the inferior frontal gyrus.

## Additional Information and Declarations

### Data Availability

The following information was supplied regarding data availability: The data is available at figshare: Chen, Xin (2023). raw data.rar. figshare. Media. https://doi.org/10.6084/m9.figshare.21936444.v1.
