# Supplementary figures and images for: The gray matter atrophy and related network changes occur in the higher cognitive region rather than the primary sensorimotor cortex after spinal cord injury
Source: PeerJ. 2023 Oct 9;11:e16172. doi: 10.7717/peerj.16172 (PMC10569206; doi:10.7717/peerj.16172)

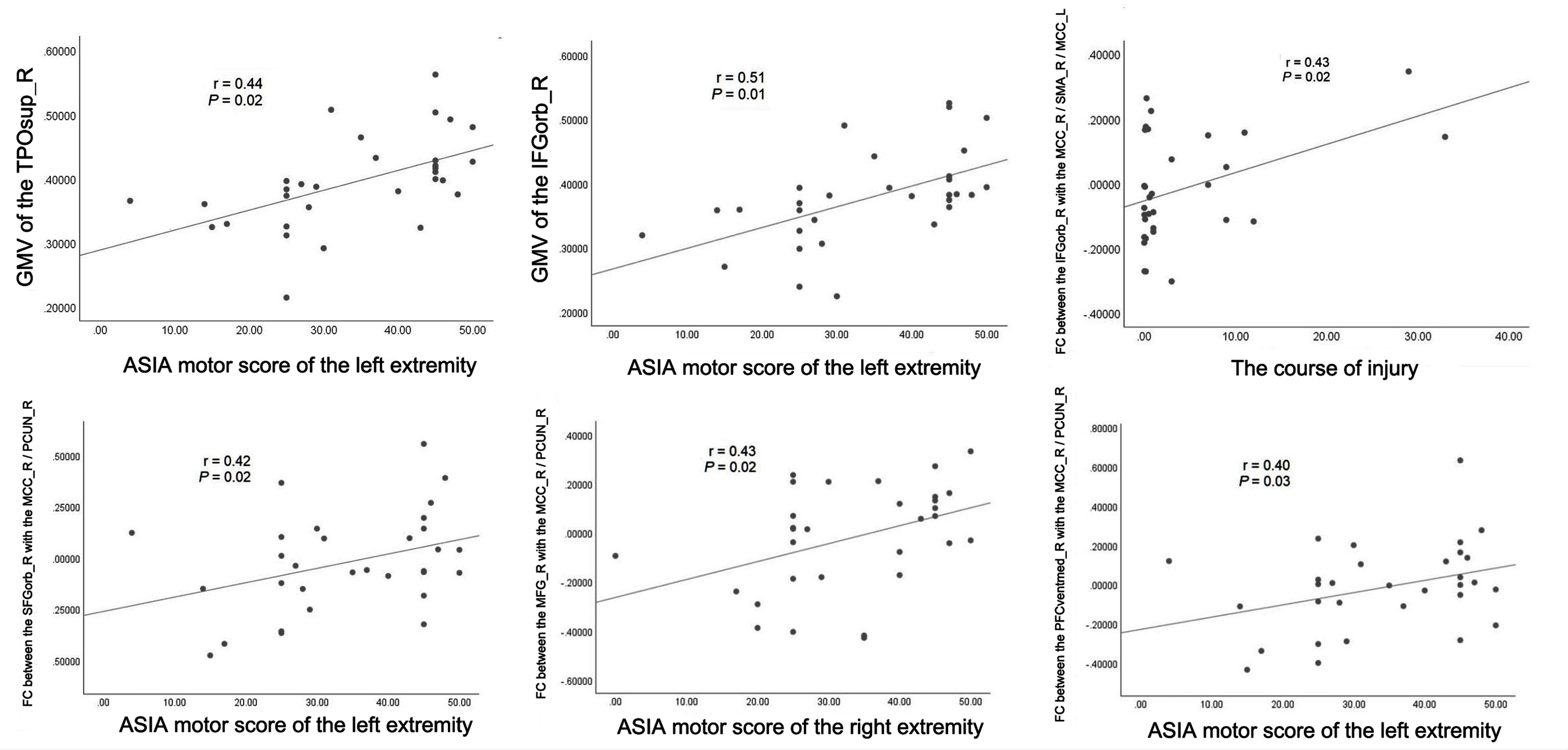

Supplement: Figure S1 — L, left; R, right; SMA, supplementary motor area; IFGorb, pars orbitalis in the inferior frontal gyrus; TPOsup, pars orbitalis in the inferior frontal gyrus; MCC, middle cingulate; SFGorb, pars orbitalis in the superior frontal gyrus; MFG, middle frontal gyrus; and PFCventmed, medial orbital in the superior frontal gyrus. [file peerj-11-16172-s001.png]
