# Supplementary material for: The gray matter atrophy and related network changes occur in the higher cognitive region rather than the primary sensorimotor cortex after spinal cord injury
Source: PeerJ. 2023 Oct 9;11:e16172. doi: 10.7717/peerj.16172 (PMC10569206; doi:10.7717/peerj.16172)
Supplement: Table S1 — Note: ASIA, American Spinal Injury Association; L, left; R, right; MFG, middle frontal gyrus; MCC, middle cingulate; INS, insular; and IFGorb, the pars orbitalis in the inferior frontal gyrus. [file peerj-11-16172-s002.docx]

**Table S1** Correlation between GMV and FC values and clinical variables in the SCI subgroups that showed statistical differences in GMV.

|  | Group | Side of the ASIA motor score | *r* | *p* |
| --- | --- | --- | --- | --- |
| FC between the sub-cluster MFG_R with MCC_R / precuneus_R | Entire group | Right | 0.43 | 0.02 |
|  | Complete sub-group | Left | 0.62 | 0.04 |
|  |  | Right | 0.72 | 0.01 |
|  | Incomplete sub-group | Right | 0.58 | 0.01 |
| Sub-cluster INS_L | Entire group | None |  |  |
|  | Pain sub-group | Left | 0.64 | 0.01 |
|  |  | Right | 0.61 | 0.01 |
|  | Pain-free sub-group | None |  |  |
| Sub-cluster IFGorb_L | Entire group | None |  |  |
|  | Pain sub-group | Left | 0.60 | 0.01 |
|  |  | Right | 0.64 | 0.01 |
|  | Pain-free sub-group | None |  |  |

Note: ASIA, American Spinal Injury Association; L, left; R, right; MFG, middle frontal gyrus; MCC, middle cingulate; INS, insular; and IFGorb, the pars orbitalis in the inferior frontal gyrus.
